# The Effects of Aspirin dose in Children with Congenital and Acquired Heart Disease. Results from the Paediatric Study of Aspirin Efficacy using Diagnostic and Monitoring Tools (PAED-M)

**DOI:** 10.1007/s00246-024-03509-6

**Published:** 2024-05-16

**Authors:** Irene E. Regan, Dermot Cox, Sean T. Kelleher, Colin J. McMahon

**Affiliations:** 1 Department of Coagulation/Haematology, Children’s Health Ireland at Crumlin, Dublin, Ireland; 2 School of Pharmacy and Biomolecular Sciences, Royal College of Surgeons Ireland, Dublin, Ireland; 3 Department of Paediatric Cardiology, Children’s Health Ireland at Crumlin, Dublin, Ireland; 4 School of Medicine, University College Dublin, Belfield, Dublin 4, Ireland; 5 School of Health Professions Education (SHE), Maastricht University, Maastricht, Netherlands; 6 National Children’s Research Centre, Children’s Health Ireland, Dublin, Ireland

**Keywords:** Aspirin dose, Congenital heart disease, Thrombosis, Resistance

## Abstract

The optimal dose of aspirin required in children with congenital and acquired heart disease is not known. The primary aim of this prospective observational study was to evaluate the effects of aspirin dose on platelet inhibition. The secondary aim was to determine the prevalence and clinical predictors of aspirin non-responsiveness. Measurements were by Thromboelastography with Platelet Mapping (TEGPM) only in children less than 2 years (y) of age with particular emphasis on the parameter known as maximum amplitude with arachidonic acid (MAAA) and using both TEGPM, and light transmission aggregometry (LTA) in children greater than 2 y. We prospectively studied 101 patients with congenital and acquired cardiac disease who were receiving empirical doses of aspirin for a minimum of 4 weeks but no other antiplatelet agents. Patients were stratified according to dose concentration and age. There was a trend toward lower age in patients with no response or semi-response to aspirin. All patients were considered responsive to aspirin in the higher-dose quartile (Q4) with a median dose of 4.72 (4.18–6.05) mg/kg/day suggesting that patients in this age group may require 5 mg/kg/day as an empirical dose. In children > 2 y, there was no significant difference in inhibition found in patients dosed at higher doses in Q3 versus Q4 suggesting that patients in this cohort are responsive with 3 mg/kg/day dose. The current practices may lead to reduced platelet inhibition in some children due to under-dosing or overdosing in others. In conclusion, younger children require higher doses of aspirin. Laboratory assessment is warranted in this population to mitigate against under and overdosing.

## Background

Aspirin has wide-ranging indications for infants and children with congenital and acquired heart disease including those with single-ventricle physiology post-systemic to pulmonary artery shunt, those with intracardiac stents or devices, and Kawasaki disease who are likewise at risk for thrombotic events, including shunt thrombosis, coronary artery thrombosis, and thromboembolic arterial stroke [1]. While the benefits of aspirin are undisputed, it is also recognized that there is a dose-dependent risk of bleeding associated with the drug [2].

Antithrombotic regimens vary across institutions but most pediatric cardiology centers administer aspirin following procedures that employ prosthetic materials such as shunts, conduits, grafts, and valves, and/or demonstrate low-velocity circulation [3]. While aspirin is often used in children with these conditions, as in adults [4, 5] there is also much debate surrounding the relationship of aspirin dose to treatment and data guiding aspirin dosing in children to clarify these recommendations are limited [2, 5]. Optimal pediatric doses of aspirin are not based on any clinical trials to date. Most treatment guidelines recommend the lowest effective dose for the prevention of thrombotic complications and to mitigate the risk of major bleeding [3, 6]. The dose of aspirin required for inhibition of platelet aggregation in pediatric patients is unknown to date, however empiric low doses of 1–5 mg/kg/day have been proposed by the CHEST guidelines [3] and supported by the Scientific Statement from the American Heart Association [6]. A single cohort study in neonates by Mir and colleagues suggests the empirical use of 40 mg/day as thromboprophylaxis if aspirin resistance is evident after 5 days on 5 mg/kg [7]. A more recent study from Emani and colleagues supports this finding and their dosing for neonates as a consequence changed to 40.5 mg/day [8]. The current guidelines on antithrombotic therapy in neonates and children also do not specifically recommend the use of specific aggregation tests to monitor or personalize aspirin therapy [3]. Nevertheless, several studies have been conducted in recent years in pediatric patients treated with aspirin following cardiac surgery to assess the prevalence of poor response to aspirin [8, 9]. Much of the published literature on aspirin responsiveness in the pediatric population has been performed in the postoperative period [7, 8, 10] which is a well-recognized high-risk period for thrombotic events with comparatively little on stable patients managed in outpatient settings who remain at risk of thromboembolic events [11].

Aspirin doses of 75 mg/day in adults are typically sufficient to inhibit platelet aggregation using in vitro testing [12]. Although the appropriate doses of antithrombotic drugs in children are known to differ substantially from adults [3], the appropriate dose of aspirin in children is unknown. In the present study, we, therefore, sought to determine in infants and children at risk for arterial thrombosis the appropriate dose of aspirin that would achieve similar levels of inhibition as 75 mg in adults as measured using two in vitro tests; the Thromboelastography with Platelet Mapping (TEGPM) parameter known as maximum amplitude with arachidonic acid (MAAA) and arachidonic acid-induced light transmission aggregometry (LTA-AA).We have found in a previous study that the MAAA is a useful marker for monitoring aspirin response which also significantly reduces blood sample requirements [13].

The aim of this prospective observational study in patients on long-term aspirin was to evaluate the effect of dose on platelet inhibition by aspirin and to determine if aspirin administered to children attending cardiology outpatient clinics resulted in adequate aspirin response. The secondary aim was to determine the prevalence and clinical predictors of non-responsiveness or poor response to aspirin.

## Materials and Methods

### Study Design

The study conforms with the principles outlined in the Declaration of Helsinki and was approved by The Research and Ethics Committees of the Children’s Health Ireland at Crumlin, Dublin, Ireland. All subjects were children and infants attending the National Cardiac Center, Children’s Health Ireland at Crumlin, Dublin, Ireland between January 2019 and December 2022. Parental consent and assent (where applicable) were received for all participants. The treating clinicians were blinded to the results of the study and the dose of aspirin was not adjusted for the purposes of this investigation. As patients were already under aspirin treatment on enrollment and the discontinuation of aspirin treatment to get a baseline was deemed unethical the control cohort was used to derive mean values for each of the parameters and the patient results were adjusted accordingly. The control cohort was also compared with the patient group on aspirin. The most specific tests for the effect of aspirin are directly dependent on COX-1 [14, 15]; hence, laboratory assays included gold standard methods (LTA-AA, and TXB2) and a modification of platelet aggregation which has the benefit of providing information on hemostatic status (TEGPM).

### Patients and Healthy Subjects

Patients eligible for enrollment in the study were ≤ 18 y with congenital or acquired heart disease requiring aspirin therapy. We consecutively enrolled patients attending outpatient clinics (*n* = 105) who were receiving aspirin at an empirical concentration of 1–5 mg/kg/day up to a maximum dose of 75 mg/day for a minimum of 4 weeks but no other antiplatelet agents**.** Due to ethical considerations in stopping treatment to get baseline results and because of assumed age-related differences in platelet parameters a healthy age-matched cohort was used to calculate the approximate inhibitory effect of aspirin. This cohort was also used to determine typically normal results using the different platelet assays of patients not taking aspirin. Healthy age-matched children attending the innocent heart murmur clinics confirmed with normal cardiovascular status served as a control group. This control group stratified into six age categories with 20 patients in each (*n* = 120) was not taking any drugs known to affect platelet function. To avoid any interference from conditions known to affect platelet production, we excluded patients (in both groups) with malignant or hematological disease or thrombocytopenia. Patients on dual antiplatelet therapies or other medications such as NSAIDs, or prostaglandins known to affect platelet function, or anticoagulants, or had undergone major surgery within 3 weeks of enrollment were also excluded. Patients were stratified into four sequential quartiles based on their aspirin dose. Patients and controls were also stratified by age to adjust their dose responses; infants aged 1 to 3 months, children aged 3 months to less than 2 y, 2 to 5 y, 5 to 10 y, 10 to 14 y, and 14 to 18 y.

### Clinical Data Collection

Patient demographic and clinical data collected at the outpatient clinic visits included cardiac diagnosis, most recent surgical procedure, gender, date of birth, weight, genetic syndrome, medications, and patient history of thrombosis or bleeding. Data also included aspirin dose, parental report of missed aspirin doses within the prior 7 days, and time of last aspirin dose. All patients also had a full blood count (FBC), coagulation screen, C-reactive protein (CRP), and a recent echocardiogram.

### Thromboelastography with Platelet Mapping (TEGPM)

A Platelet Mapping assay (TEGPM) using the TEG^®^ 5000 analyzer was used to evaluate platelet aggregation and inhibition according to the manufacturer’s instructions. TEGPM is a modification of the TEG assay that isolates platelet function in the clotting process and allows for the comparison of the maximal amplitude (MA) value, under three different conditions in the case of aspirin. The percentage of platelet inhibition using the arachidonic acid (AA) agonist is calculated by TEGPM software as [100 − {(MAAA –MAFibrin)/(MAThrombin–MAFibrin) × 100}]. The same software was also employed to calculate the percentage residual platelet aggregation as 100-platelet inhibition (%). All the above TEGPM parameters including the maximal amplitude (MAAA), percentage platelet inhibition, and aggregation were assessed and recorded on all subjects. The MAAA parameter was evaluated as a marker for use in preference to the overall % inhibition calculation using both citrate, and heparin samples. The TEG MAAA can be described as the maximal platelet activation by the thromboxane A2 pathway and is measured using a lithium heparin whole blood sample activated with reptilase, FXIIIa, and arachidonic acid (AA). This test measures residual platelet activity in the presence of aspirin. Patient results were adjusted using the mean control values for the appropriate age using the following formula:

Percentage inhibition corrected for age = ((Control MeanMAAA − PatientMAAA)/Control MeanMAAA) × 100. Blood samples were analyzed within 2.5 h after collection.

### Platelet Light Transmission Aggregometry (LTA)

Due to ethical considerations for blood volume, only patients ≥ 2 y had LTA performed alongside the TEGPM. Platelet aggregation studies were performed within 4 h of blood collection in 3.2% citrate [16]. The percentage inhibition for each patient was calculated using the mean control aggregation for each agonist as a baseline. Agonists included arachidonic acid (1 mM) and collagen (2ug/ml).

### Serum Thromboxane B2

Thromboxane B2 is the stable metabolite of thromboxane A2. All platelet aggregation/inhibition results were correlated with serum thromboxane B2 results to measure adherence to aspirin [17].

### Aspirin Non-response (Resistance) Definitions

There are no manufacturer-recommended ranges for any of the parameters or cut-offs for aspirin response or definitions regarding the effect of drugs on the MA. We defined patients as having inadequate response to aspirin as those with inhibition of TEG (MAAA) of < 50% when the patients’ MA was adjusted using age-appropriate control results. Adult studies and some pediatric studies define a response to the drug as > 50% inhibition of the MA, a partial response as 30–50% inhibition, and a lack of response as less than 30% inhibition when compared with kaolin heparinase TEGPM-MA [5]. Other studies defined resistance as platelet aggregation > 20% using arachidonic acid [18]. We defined this aggregation cut-off of 20% as equivalent to 75% inhibition from a mean control of approximately 85% (control results). The laboratory criteria applied in this study for aspirin non-response was platelet inhibition < 50%, semi-response 20–50% inhibition by TEGPM, and LTA-AA (in patients > 2 y) > 20% or age-adjusted inhibition of aggregation < 75%. A category of semi-response was not applied in the case of LTA measurements. TEGPM data were confirmed using a surrogate AA (Helena Biosciences). A cut-off > 40 mm for MAAA was also used as quality control verification.

### Statistical Analysis

All samples were measured in duplicate, and results were only reported when consistent with each other. Repeated samples were analyzed on any patients showing reduced response to aspirin. The initial and repeated samples were spiked ex vivo with aspirin to investigate responsiveness by reanalysing the MAAA. Patients and controls were stratified according to age to account for variations associated with age. Patients were further stratified into aspirin dose quartiles. Normally distributed data are expressed as mean ± SD, and non-parametric data are expressed as median (range). Categorical variables are reported as percentages and were compared using Pearson’s chi-squared test or Fischer’s exact test. ANOVA was also used to analyze differences between quartiles; a Kruskal–Wallis ANOVA was used if the data were not normally distributed. All hypothesis tests were performed with 2-sided tests. Pearson’s correlation coefficient was calculated for the relationship between dose concentration and each of the platelet inhibition and aggregation parameters. All statistical analyses were performed using SPSS version 26 (IBM Corporation, New York, NY, United States of America) with a *p*-value < 0.05 considered statistically significant. Results are summarized by dose and age group.

## Results

### Patient Demographics and Laboratory Characteristics

A total of 105 children who met the eligibility criteria were included in the study and had complete data. Four patients (3.8%) were then excluded due to high TXB2 levels consistent with non-adherence to aspirin, confirmed on follow-up discussions with the relevant parents. The characteristics of this study population (*n* = 101) are summarized in Table 1. The median age was 3549 days with a range of 56–6652 days, 58 girls, and 43 boys. The most common cardiac diagnoses were as follows: hypoplastic left heart syndrome in 45.5%, Tetralogy of Fallot (8.9%), and cardiomyopathy in 8.9%. A total of 50 patients (49.5%) patients followed the single-ventricle surgical pathway, 31.7% had an intracardiac or intravascular stent placed, 5.9% had Kawasaki disease, 8.9% had cardiomyopathy, and the remaining patients had an arterial graft in place. All patients were taking aspirin at the empirical dose.

**Table 1:** Demographic characteristics of aspirin-treated patients stratified into dose quartiles

	Aspirin dose mg/kg/day				Overall	*p* value
	Q1	Q2	Q3	Q4		
*Characteristic*	1.12–1.97	2.03- 2.91	3.0–3.91	4.17–6.05		< 0.05
No. of patients	26	25	25	25	101	0.95
Median Age	4759 (2388–6652)	3718 (139–6216)	1096 (125–3535)	1156 (56–4392)	3549 (56—6652)	< 0.05
Females, % patients	50	64	56	60	57.4	0.48
Weight (kg)	50.9(25.0–66.6)	31.0 (6.58- 36.8)	11.6 (5.36- 24.4)	12.0 (4.0- 18.0)	29 (4.0–66.6)	< 0.05
Dose (mg/kg/day)	1.44 (1.12–1.97)	2.34 (2.03–2.91)	3.37 (3.0- 3.91)	4.69 (4.17- 6.05)	2.34 (1.12–6.05)	< 0.05
*Diagnosis, % patients*						
Hypoplastic left heart syndrome variants	57.7	32	48	44	45.5	
Tetralogy of fallot	11.5	8	0	16	8.9	
Cardiomyopathy	0	24	8	4	8.9	
Kawasaki	0	12	8	4	5.9	
Ventricular septal defects	0	8	4	8	4.9	
Pulmonary atresia with VSD/AVSD	0	0	8	8	4	
Pulmonary atresia, VSD, MAPCAS	0	0	8	4	3	
Aortic valve disease	3.8	4	4	0	3	
Truncus arteriosus	0	0	4	8	3	
Pulmonary atresia with intact ventricular septum	3.8	0	4	0	2	
Unbalanced AVSD with hypoplastic RV/LV	3.8	0	4	0	2	
Transposition of the great arteries	3.8	4	0	0	2	
Patent ductus arteriosus	7.6	0	0	0	2	
Coarctation of the aorta	7.6	0	0	0	2	
Left ventricular diverticulum	0	4	0	0	1	
Tricuspid atresia	0	0	0	4	1	
Arterial calcification	0	4	0	0	1	
*Most recent surgery, %*						
Fontan	57.1	36.8	32.1	33.3	40.2	
Glenn	3.6	15.8	14.3	11.1	8.8	
RV-PA conduit	10.7	5.3	17.9	7.4	12.7	
Tetralogy of Fallot repair	0	10.5	10.7	0	4.9	
PDA repair	7.1	0	0	0	2	
VSD/ASD closure	0	10.5	7.1	14.8	7.8	
Ross procedure	0	5.3	3.6	0	2	
Stent placement	21.4	15.8	14.3	33.3	21.6	

A number of patients had > 1 surgery

*RV-PA* right ventricle to pulmonary artery

Patients were stratified into quartiles based on their dose of aspirin resulting in the following ranges: Q1 = 1.12–1.97 mg/kg/day, Q2 = 2.03–2.91, Q3 = 3.0–3.91, and the upper Q4 greater than 4.17 mg/kg/day with an expected overall significant difference between quartiles (ANOVA, *p* < 0.05). Similarly, there was a significant difference in age and weight across the dose quartiles with older and heavier children generally in the lower dose quartiles. There was no significant difference in hematology parameters including immature platelet fraction (a marker for platelet turnover) across the dose quartiles as shown in Table 2.

**Table 2:** Hematology parameters for the patient cohort on aspirin stratified into dose quartiles

Aspirin dose mg/kg/day						
	Q1	Q2	Q3	Q4	Overall	*p* value
	1.12–1.97	2.0–2.91	3.0–3.91	4.17–6.05		< 0.05
*Laboratory characteristics*						
No	26	25	25	25	101	0.95
Hemoglobin (g/l)	133.8 ± 20.4	132.8 ± 20.0	129.8 ± 20.9	129.7 ± 21.1	131.6 ± 21.8	0.84
HCT (%)	39.7 ± 2.9	38.4 ± 4.4	37.2 ± 5.6	38.0 ± 4.3	38.30 ± 4.15	0.61
WBC (10^9^/l)	6.93 ± 2.33	6.29 ± 2.27	8.77 ± 2.68	6.42 ± 3.62	7.10 ± 2.63	0.13
Platelets (10^9^/l)	266.3 ± 84.4	235.6 ± 65.5	299.4 ± 74.0	285.4 ± 96.2	271.6 ± 79.9	0.24
MPV (Fl)	10.93 ± 0.90	10.86 ± 0.83	10.14 ± 1.08	11.01 ± 1.87	10.76 ± 1.10	0.28
IPF (%), median	3.85 (0.8- 6.6)	2.65 (1.0- 3.2)	1.80 (0.8—3.2)	1.89 (0.6- 3.4)	2.70 (0.6- 6.6)	0.77
IPF (%), mean	3.95 ± 2.28	2.40 ± 0.80	1.76 ± 0.84	1.80 ± 0.70	2.48 ± 2.4	0.70
Ret (%)	1.64 ± 0.66	1.09 ± 0.34	1.50 ± 0.70	1.06 ± 0.40	1.33 ± 0.54	0.79

Median (range), Mean ± SD

*WBC* white cell count, *HCT* haematocrit, *MPV* mean platelet volume, *IPF* Immature platelet fraction, *Ret* reticulocyte count

### Controls

Demographics and LTA-AA, LTA-Collagen, and TEG (MAAA) measurements of the age-matched controls stratified into six age ranges are summarized (Table 3). The MAAA at a median of 67.3 mm and range 60.3–72.5 mm was significantly higher in infants < 3 months of age when compared with other age categories (*p* < 0.05). The MAAA was observed to reduce in the older age cohorts with LTA-AA and LTA-Collagen measurements consistent across the age categories. The percentage inhibition of patients on aspirin and results are displayed in Tables 4 and 5. A significant effect of aspirin treatment in patients compared with the healthy control cohort was observed (*p* < 0.05).

**Table 3:** Platelet aggregation parameters of the control cohort stratified into age ranges

Controls (*n* = 120)							
Age cohort	1–3 m	3 m–1 y 364 days)	2–5 y	5–10 y	10–14 y	14–18 y	*p* value
No	20	20	20	20	20	20	
Age, days	59(33–86)	302 (100–700)	1024 (800–1793)	2450 (1995–3470)	4503 (3780–5026)	5506 (5270–5934)	< 0.05
TEG- MAA (mm)	67.3 (60.3–72.5)	61.85 (58.1–71.3)	62.2 (55.9–69.7)	58.6 (53.3–65.9)	59.8 (52.2–67.4)	62.8 (60.5–66.5)	< 0.05
TEG-MAA (mm) mean ± SD	66.8 ± 5.6	63.2 ± 5.5	61.5 ± 6.7	59.2 ± 6.9	59.0 ± 7.1	62.9 ± 2.4	< 0.05
LTA-AA (%) median (range)			82.5 (80.3–87.2)	85.3 (78.5–90.0)	84.5 (77.3–90.5)	85.0 (75.3–89.0)	NS
LTA-AA (%) mean ± SD			81.5 ± 4.1	84.2 ± 5.5	84.0 ± 6.6	85.0 ± 5.5	NS
LTA-Coll (%) median (range)			84.9 (77.3–91.9)	87.8 (81.5–92.5)	87.0 (79.3–93.0)	86.0 (79.2–94.0)	NS
LTA-Coll (%) mean + SD			85.6 ± 7.0	86.9 ± 5.5	87.3 ± 5	87.0 ± 7.0	NS

Median (range), Mean ± SD

*NS* non-significant

**Table 4:** Aspirin response in patients less than 2 years of age

Patients less than 2 y (*n* = 44)					
	Aspirin dose mg/kg/day				*p* value
	Q1	Q2	Q3	Q4	
Quartile dose (mg/kg/day)	1.44 (1.12–1.97)	2.34 (2.03–2.91)	3.37 (3.0–3.91)	4.69 (4.17–6.05)	< 0.05
No	0	20	12	12	
Age, days		125 (56–707)	277 (125–430)	356 (304–408)	0.057
Dose (mg/kg/day)		2.78 (2.29–2.91)	3.70 (3.49–3.91)	4.72 (4.18–6.05)	< 0.05
*Method*					
Age-adjusted Inhibition by MAAA (%)		42.3 (− 23.9–60.9)	57.5 (4.3–63.9)	80.0 (69.0–93.0)	< 0.05
*Interpretation of response*		2 non-responders, 2 semi-responders	2 non-responders	All responsive	
Diagnosis: non-responders		Truncus arteriosus, TOF	Truncus arteriosus		
Surgery: non-responders		RV-PA conduit, stent	RV-PA conduit		
Diagnosis: semi-responders		HLHS			
Surgery: semi-responders		Glenn			

Medians (range)

*TEGPM MAAA*; Responder =  > 50%, semi-responder = 20–50%, non-responder < 20%

**Table 5:** Aspirin response in patients over 2 years of age

Patients greater than 2 y (n = 57)					
	Aspirin dose mg/kg/day				*p* value
	Q1	Q2	Q3	Q4	
Quartile dose (mg/kg/day)	1.44 (1.12–1.97)	2.34 (2.03–2.91)	3.37 (3.0–3.91)	4.69 (4.17—6.05)	< 0.05
No. patients	19	14	11	13	
Dose (mg/kg/day)	1.44 (1.13–1.97)	2.37 (2.03–2.88)	3.26 (3.0–3.90)	4.43 (4.17–6.01)	< 0.05
Age, days	4759 (2388 -6652)	4080 (770—6316)	1096 (788–3535)	1242 (888—4302)	< 0.05
Age-adjusted inhibition MAAA (%)	73.9 (− 26.5–100)	78.8 (33–100)	77.5 (57.5–88.3)	83.2 (67.5–95.8)	0.554
LTA-AA aggregation (%)	12.0 (0.9–36.2)	12.1 (0.9–23.5)	11.1 (5.9–12.5)	9.3 (4.8–18.1)	0.544
LTA-collagen aggregation (%)	57.0 (40.8–65.0)	48.0 (35.0–59.6)	41.0 (37.0–44.0)	35.0 (30.0–39.0)	< 0.05
LTA-AA % inhibition	84.2 (56.7–98.9)	84.1 (69.1–98.9)	85.4 (85.1–92.9)	88.9 (76.4–94.3)	0.533
LTA-Coll % Inhibition	34.2 (25.0–52.9)	44.6 (36.6–59.6)	52.7 (48.1–57.3)	59.6 (55.0–65.4)	< 0.05
*Interpretation of overall response*					
MAAA	2 non-responders, 2 semi	2 semi-responders	All responsive	All responsive	
Diagnosis: non-responders	TGA, HLHS	HLHS × 2			
Surgery: non-responders	RV-PA conduit, Fontan	Glenn, Fontan			
Diagnosis: semi-responders	HLHS × 2				
Surgery: semi-responders	Glenn, Fontan				
LTA-AA % Inhibition	4 Non-responders	2 Non-responders	Same	Same	

Medians (range)

TEGPM MAAA; Responder, > 50% Inhibition MAAAA, semi-responder, 20–50%, non-responder < 20%. LTA-AA% Inhibition; Responder, > 75%, no criteria for semi-response

### Dose Response

**Fig. 1:**
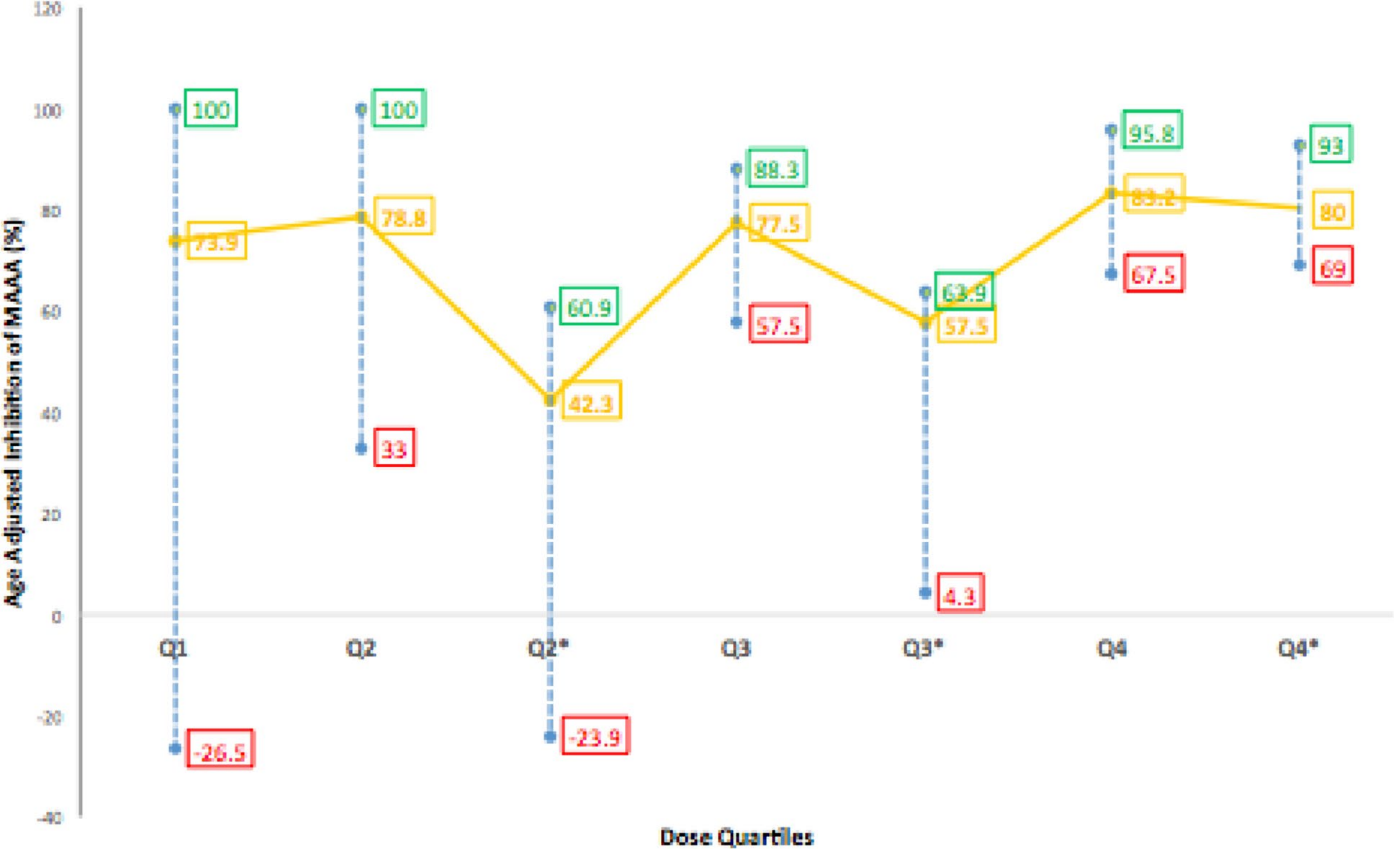
Effects of aspirin on AA-induced platelet aggregation in children less than, greater than 2 years of age using TEGPM, and marker MAAA. Graph demonstrates the age-adjusted percent inhibition at each dose quartile (median, range). Q1, Q2, Q3, and Q4 indicate dose quartiles for patients > 2 years of age. Q2*, Q3*, and Q4*indicate dose quartiles for patients < 2 years of age

Each MAAA result was adjusted according to age using the mean control results relevant to its age category (Fig. 1). Patients on aspirin were divided into two age groups: < 2 (*n* = 44) and > 2 (*n* = 57) years of age (Table 4, 5). In general, the majority of patients, which included wide variation in age, diagnoses, surgery, and shunt type responded well to aspirin. However, thirteen patients (12.9%) had a reduced response to aspirin.

In the patients < 2 y, there were no patients in the first quartile (Q1). In this age cohort, a total of 6 patients (13.6%) had a subtherapeutic response to aspirin, 4 patients (9.1%) were defined as non-responsive, and 2 patients were semi-responsive (4.5%). This was further defined as 2 patients in Q2 and Q3 as non-responders by TEG and LTA-cut-off criteria and 2 semi-responders in Q2. All patients were considered responsive to aspirin in the higher-dose Q4. There was wide variation in the response to aspirin across the dose quartiles with some patients reaching 63.9% inhibition in Q3 and then others having only 4.3% inhibition. All patients in the upper dose quartile (4.16–6.0 mg/kg/day) reached a statistically significant platelet inhibition cut-off of 50% when we compared Q4 with Q2 and Q3; however, there was no statistically significant difference between Q2 and Q3. There was a non-significant weak correlation between dose concentration and TEG (MAAA) inhibition (*r* = 0.173, *p* = 0.682).

**Fig. 2:**
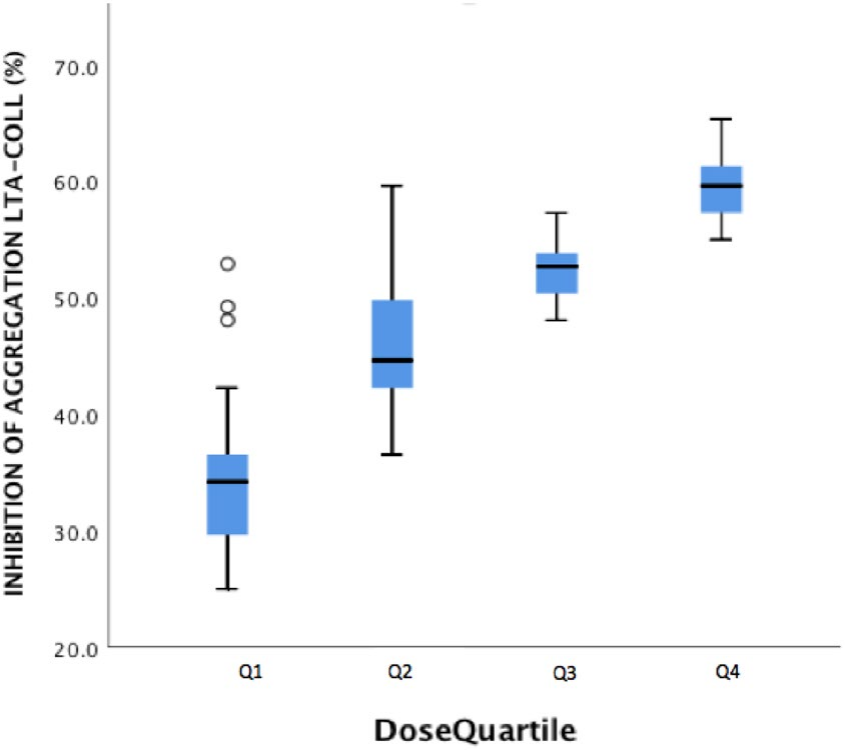
Percent Inhibition of aggregation by LTA-Collagen in patients divided into dose quartiles (Comparison of means by ANOVA *p* < 0.05, Correlation *r* = 0.821, *p* < 0.05)

In the patients ≥ 2 y, 6 patients had a sub-optimum response to aspirin (10.5%). There was also wide variation in this age cohort in the response to aspirin by TEG (MAAA) across the dose quartiles with some patients reaching 100% inhibition in Q1 and Q2 and others having no inhibition. A number of patients in Q2 could be seen as semi-responders (33% inhibition). All patients in higher-dose quartiles Q3 and Q4 reached 50% inhibition by TEG and 75% inhibition by LTA-AA. This data indicates that the median 3.26 mg/kg/day dose achieved a target of 50% inhibition of MAAA and higher doses were of insignificance in this age cohort. This assumption is confirmed with the LTA-AA assay where all patients in Q3 and Q4 had inhibition of ≥ 75%. Similar to patients < 2 y, patients that were semi-responsive by MAAA criteria were classified as non-responsive by LTA-AA due to classification criteria. Consistent with MAAA results, there was no significant difference in results for LTA-AA between Q3 and Q4. Similar to the patients < 2 y no correlation was found between dose concentration and MAAA (*r* = 0.079, *p* = 564). While no correlation was found between inhibition using LTA-AA and dose concentration (*r* = 0.056, *p* = 0.679), a strong correlation was found between dose concentration and inhibition using LTA-Collagen (*r* = 0.821, *p* < 0.05) as illustrated in Fig. 2. A mean aggregation cut-off of 42.5% (± 6.9) by LTA-Collagen as shown in Q3 was observed to be equivalent to MAAA and LTA-AA recommended cut-offs. The majority of patients that were prescribed 75 mg/day were taking enteric-coated preparations; however, we did not observe this to be a factor in non-responsiveness.

All patients showed response to ex vivo spiking of aspirin suggesting that a higher dose of aspirin was required in these patients. However as stated above, we did not find any overall association between the inhibition pattern using AA and aspirin dose. Duration between the last aspirin dose and blood draw were not associated with the degree of inhibition in any of the cohorts. In summary, there was marked inter-patient variability in the degree of platelet aggregation inhibition within each treatment dose group and age group.

The secondary aim was to determine the clinical predictors of aspirin resistance. In the cohort < 2 y, in Q2, the non-responders had a primary diagnosis of Truncus arteriosus, and Tetralogy of Fallot and their most recent surgeries or cardiac catheter procedures were insertion of Right ventricle to pulmonary artery (RV-PA) conduit and stent placement. The two patients who were classified as semi-responsive had a primary diagnosis of hypoplastic left heart syndrome and their most recent surgery being a Glenn procedure. In Q3, both patients that were non-responsive to aspirin had a primary diagnosis of Truncus arteriosus and a RV-PA conduit procedure. In the cohort ≥ 2 y of age in Q1, the first patient who was non-responsive had a primary diagnosis of Transposition of the great arteries and a RV-PA conduit and the second patient was diagnosed with hypoplastic left heart syndrome and had a Fontan procedure. Both semi-responsive patients had a primary diagnosis of hypoplastic left heart with one patient post-Glenn and the remaining patient following the Fontan procedures. In Q2 two patients were semi-responsive to aspirin, both with a primary diagnosis of hypoplastic left heart, one post-Glenn, and the other post-Fontan. In summary, although the numbers are small, all patients who had a Glenn procedure were semi-responsive to aspirin in Q1 and Q2 and responsive at the higher doses, 30% of patients who had a RV-PA conduit were classified as unresponsive. All patients with a diagnosis of cardiomyopathy or Kawasaki disease were responsive to aspirin at all doses. We could however find no overall association between the primary diagnosis or surgery and responsiveness to aspirin.

## Discussion

The present prospective observational study assessed platelet responsiveness to various empirical aspirin doses by commonly used assays in pediatric cardiology patients. Firstly, we cannot ignore that four of the patients (3.8%) were found to be non-adherent to aspirin. While adherence to drug schedules is assumed to be better in children than adults there is a paucity of evidence in this area. Gencheva et al. in their meta-analysis of 10 antiplatelet studies found that non-compliance in adult patients ranges from 12 to 52% with only 3–21% due to drug-induced adverse events, an important finding when interpreting results of trials and studies [19].

The data illustrate that there is variability in the percentage inhibition of platelet aggregation with aspirin. The present data show that children < 2 y require a higher dose of aspirin per kilogram of body weight than older children to achieve 50% inhibition of TEG (MAAA) and 75% inhibition of LTA-AA, i.e., the levels of inhibition achieved with the approved adult dose of aspirin. This finding is consistent with previous studies [7, 8] which supports a more strategic stance to testing followed by dose adjustment if unresponsive [7, 8]. All patients were considered responsive to aspirin in the higher quartile (Q4) with a median dose of 4.72 (4.18–6.05) mg/kg/day suggesting that patients in this age group may require 5 mg/kg/day as an empirical dose.

In children ≥ 2 y, there was no significant difference in inhibition found in patients dosed at higher doses in Q3 versus Q4. We did not find any association between platelet turnover and inhibition or duration between the last aspirin dose and blood draw. The majority of patients that were prescribed 75 mg/day were taking enteric-coated preparations which is known to have significant effects on bio-availability when studied in adults [17, 20, 21] and this is the norm unless specified by the prescribing physician. We however did not observe this to be a factor in non-responsiveness in this study. All patients in higher quartiles Q3 and Q4 reached 50% inhibition by TEG and 75% inhibition by LTA-AA. This data indicates that the median 3.26 and range (3.0–3.9) mg/kg/day dose achieved a target of 50% inhibition of MAAA and higher doses were of insignificance in this age cohort. This data suggests that patients ≥ 2 y only require 3 mg/kg/day. This may however present a dilemma for children who are on a maximum dose of 75 mg/day, suggesting that such patients would require initial assessment for responsiveness with a view to altering the maximum dose, changing therapy, or initiation of dual therapy.

Due to low numbers, it was difficult to find a clear association between surgery, diagnosis, and response to aspirin. We did however observe that all patients post-Glenn were semi-responsive to aspirin in lower quartiles (Q1, Q2) and had satisfactory platelet inhibition in Q3 and Q4 suggesting that these patients as a group may require more aspirin. While these patients may be at a lower risk for thrombosis than at other stages of the single-ventricle palliation pathway, the importance of aspirin in the prevention of chronic sub-clinical thromboembolic events which may be detrimental to the pulmonary vascular bed is recognized. Li et al. in a prospective multicenter observational study concluded that aspirin appears to lower the risk of thrombosis and improve overall survival in shunt-dependent patients [22] and is therefore of importance that aspirin effectiveness is maintained. 30% of our study patients with a RV-PA conduit were classified as unresponsive to aspirin, a possible risk factor for further consequences as previously described [23, 24]. Malekzadeh-Milani et al*.* found that the use of antiplatelets reduced the need for emergency valve replacement and overall mortality. Habib et al. reported that aspirin thromboprophylaxis before the onset of cardiovascular implantable electronic device-related bloodstream infection was associated with a lower likelihood of vegetation formation on the device [23] and in a similar manner, Eisen et al*.* concluded that aspirin usage was associated with a reduced risk of emergency valve replacement surgery in patients with Staphylococcus aureus-associated infective endocarditis [25]. As a result of the high prevalence of reduced response to aspirin in this sub-cohort, patients with RV-PA conduits were followed up in a subsequent institutional study [26]. Three patients (12.5%) in Q1 and Q2 (*n* = 3/24) had sub-optimum responses to aspirin had Fontan conduits. This is lower than the 50% poor response observed by Patregnani et al*.* [27] however, their study was conducted in the early postoperative period where the platelet turnover is high, a phenomenon thought to have an influence on platelet inhibition [10, 28]. We are in agreement with Patregnani that this sub-population of patients requires monitoring at intervals.

The ability to assess aspirin responsiveness offers great potential. All patients that were non-responsive or semi-responsive by TEGPM (full profile) or TEG (MAAA) were found to have an impaired response by LTA-AA. This novel application of the MAAA assay has the advantage of providing quantitative data and also requires only 1.2-mls blood for monitoring or repeat analysis [13]. Good agreement between methodologies that use AA as an agonist has been reported [5]. There is debate around the effect and cut-off for collagen-induced platelet aggregation by LTA, Gurbel quotes ≥ 70% in the ASPECT adult study [5], whereas other authors suggest 60% for adult studies [29]. We observed that aspirin does indeed have an inhibitory effect on collagen and is dose concentration related. Our findings were however lower than the quoted cut-offs of 70 or 60%. We observed that a mean aggregation cut-off of 42.5 ± 6.9 and mean inhibition of 49.7% ± 8 was equivalent to the effect observed in LTA-AA and TEG (MAAA) assays consistent with Santilli et al*.* [30].

The present study has some limitations. Firstly, due to ethical considerations in stopping aspirin to get a baseline, a control population was used as a baseline for the maximum aggregation; however, this population was age matched and may be a better indicator of a baseline than stopping aspirin. Although this was a relatively small group in terms of the number of patients with evaluable platelet aggregation and inhibition data in each dose group, all analyses were performed in duplicate and confirmed with repeat analysis if required before any conclusions were made. Due to sample size dose quartiles were not statistically compared with regard to age and diagnoses or surgeries. Because of ethical considerations, we did not assess LTA in patients < 2 y; however, we have confirmed our data in the older cohort using three assays. The benefits of aspirin are undisputed with a greater than seven-fold reduction in the risk of shunt thrombosis and a decreased risk of death [22]. Studies have also reported postoperative thrombosis associated with failure to respond to aspirin, with non-responders more likely to have a thrombotic event than responders [7, 31]. While this study aimed to evaluate the effect of dose rather than outcomes a follow-up prospective study will endeavor to answer whether insufficient antiplatelet inhibition is of any clinical importance in children or if it is solely a laboratory finding.

**Fig. 3:**
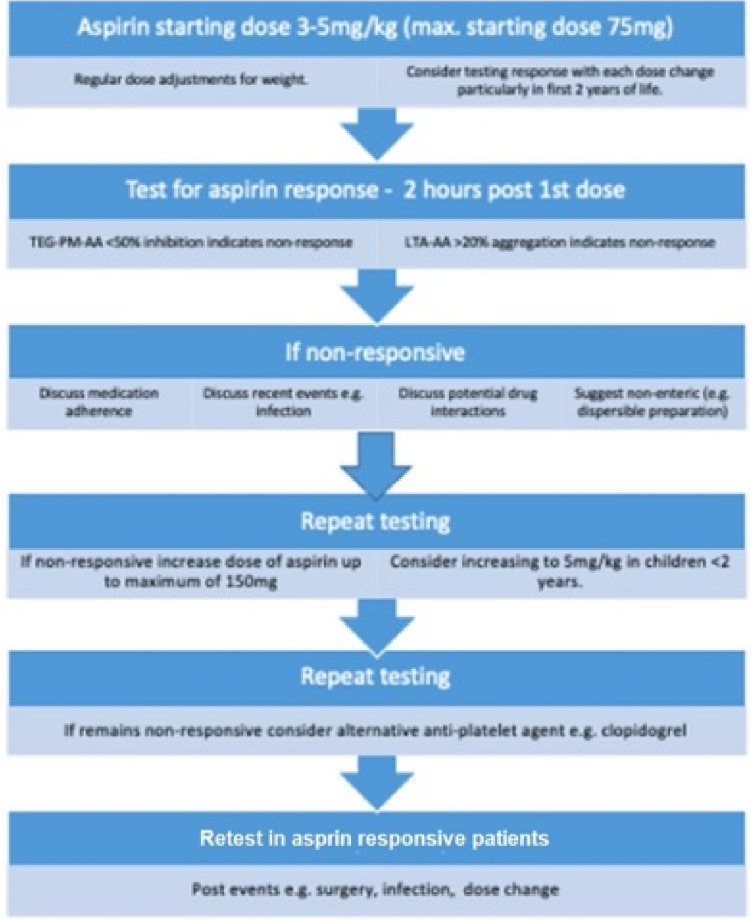
Proposed aspirin responsiveness testing algorithm[26]

We have demonstrated that individual variation in aspirin responsiveness can be measured with robust laboratory assays and effective dose adjustment may mitigate the risk of thrombosis or bleeding as found in previous studies. A proposed algorithm for testing is outlined in Fig. 3, adapted from our earlier study [26]. There has been much debate on the relationship between aspirin dose and treatment effect, particularly in adult populations [32, 33] but more recently in pediatric patients [7, 8]. We have concluded that children < 2 y require a higher dose of aspirin than older children and require at least a baseline assessment to ascertain this. In general, patients on long-term aspirin are responsive to aspirin; however, there is wide variability even at the same doses and laboratory assessment is warranted in this population to mitigate against under and overdosing.
